# *Chironomus sancticaroli* generation test: A new methodology with a Brazilian endemic insect

**DOI:** 10.1016/j.mex.2018.12.013

**Published:** 2018-12-26

**Authors:** Aline C. Bernegossi, Bruna N.P. Cardoso, Mayara C. Felipe, Mara R. de Lima e Silva, Juliano J. Corbi

**Affiliations:** Department of Hydraulic Engineering and Sanitation (SHS), School of Engineering of São Carlos, University of Sao Paulo – USP, C.P. 359, CEP 13566-590, São Carlos, SP, Brazil

**Keywords:** *Chironomus sancticaroli* generation test, *Chironomus xanthus*, Long-term ecotoxicological tests, Life cycle

## Abstract

This paper presents a new ecotoxicological test to investigate the response of a Brazilian endemic insect *Chironomus sancticaroli* through its life cycle and its future generations. This test can evaluate differences between the endpoints analyzed in diverse generations, describing the long-term impact of a substance or matrix effects along the time of exposure. Despite earlier papers already present the generation test with *Chironomus riparius*, there are still no studies with long-term test applied to *C. sancticaroli*. In this sense, this study evaluated different conditions for the development of a methodology that prolonged the duration of the test and allowed the best sampling of the organism on environmental toxicity tests. The distinct conditions tested were: volumes of test solution, frequency of feed, number of larvae, type of vessel and test solution replacement. The best condition for the *C. sancticaroli* generation test includes the exposition of 20 larvae to 340 or 500 mL of test solution, 60 g formulated sediment, feeding with Tetramin® each 10 days and, from a spawn, a new test will be prepared with the same characteristics of the previous one. This new methodology can reveal toxic effects along the exposure time and brings advances on toxicology area.

• Prolonged testing makes it possible to analyze the long-term effects
• The methodology "*C. sancticaroli* generation test" allows to evaluate the responses of organisms in different life cycles
• The methodology allows to analyze the effects of substances in liquid medium and environmental quality through exposure to sediment

## Graphical abstract

**Figure:**
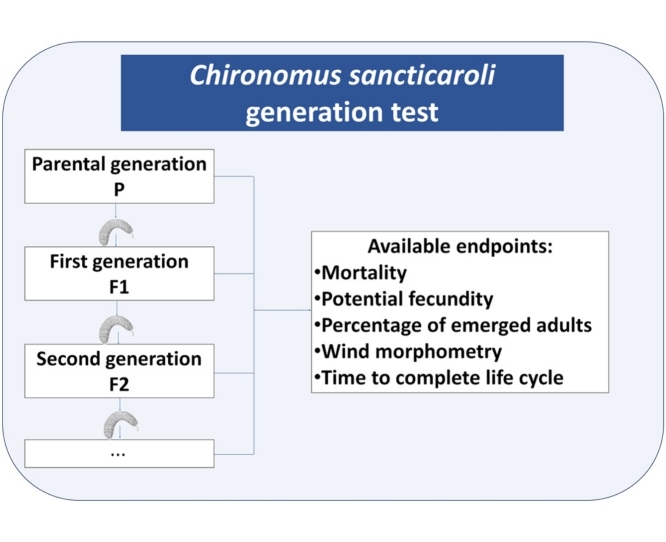

**Specifications Table**

**Table:** 

**Subject Area**	• Environmental Science
**More specific subject area:**	Aquatic Ecotoxicology
**Method name:**	*Chironomus sancticaroli* generation test
**Name and reference of original method**	**Sediment-Water Chironomid Life-Cycle Toxicity Test, In:** OECD (2010), Test No. 233: Sediment-Water Chironomid Life-Cycle Toxicity Test Using Spiked Water or Spiked Sediment, OECD Guidelines for the Testing of Chemicals, Section 2, OECD Publishing, Paris, https://doi.org/10.1787/9789264090910-en.
**Resource availability**	Not applicable

## Method details

Many authors have been utilizing *Chironomus sancticaroli* species in ecotoxicological bioassays, assessing the effects of contaminants on the development of the organisms using, for example, larvae length and adult wing length, especially for the first population generation. The present methodology, of population generation tests, can aid the researchers to obtain more detailed results in laboratory experiments and also may support sustainable decision-making by environmental agencies. Despite some authors have already used the idea of generation test on Chironomidae family, there is a lack of methods in the literature for the development of longer assays in Brazilian species [1,2]. In order to evaluate this gap, the present study proposes a methodology for the creation and accomplishment of long-term toxicity tests using *C. sancticaroli* (*C. xanthus* or *C. domizii*) in the laboratory, with the objective of covering more than one generation of the species and contributing to refine lethal and sublethal effects of diverse substances to the aquatic biota. This method can be useful in environmental ecotoxicological effects (water and sediment sample) of chemical substances and to analyze sublethal effects of substances in low concentration (parts per billion or parts per million).

## *C. sancticaroli* cultivation and maintenance

The cultivation and maintenance conditions of *C. sancticaroli* followed previous methodology proposed by [[3], [4], [5], [6]]. The first individuals of *C. sancticaroli* cultivated in laboratory were obtained from samples in stabilization ponds of a chicken slaughterhouse (BR-Aves) in São Carlos (São Paulo, Brazil).

Cultures of *C. sancticaroli* were kept in plastic trays (38 cm long × 33 cm wide × 6 cm high), covered by a nylon mesh support (42 cm long × 36 cm wide × 38 cm height) to prevent winged adult organisms from escaping from the growing area, cultures were maintained under constant aeration. Inside the trays there were 0.6 cm of sand treated as substrate and deionized water under the following conditions: conductivity between 25–55 μS cm^−1^, hardness between 12 and 16 mg L^−1^ for CaCO_3_, pH between 6.5 and 7.5, temperature of 25 ± 2 °C and photoperiod of 12 h light/ 12 h dark [7,8]. The feeding comprised the addition of macerated solid fish (Tetramin®), offered every 7 days, (5.75 mg L^-1^ of final concentration).

## Method validation

The organisms cultivated in laboratory were used to evaluate the best condition to proceed the *C. sancticaroli* generation test. This long-term ecotoxicological tests were carried out involving the entire life cycle of the *C. sancticaroli* species. The conditions tested are described in Table 1. All the parameters were described for each replicate.

**Table 1:** The conditions tested and used to evaluate the best condition to proceed the *C. sancticaroli* generation test in six assays.

Conditions tested	Assay: 1	Assay: 2	Assay: 3	Assay: 4	Assay: 5	Assay: 6
Beakers volume/ material	1,000 mL/glass	1,000 mL/glass	1,000 mL/glass	500 mL/plastic	500 mL/plastic	500 mL/plastic or 1,000 mL/glass
Beaker lock	Tulle netting	Tulle netting	Tulle netting	plastic lid	plastic lid	plastic lid or Tulle netting
Parental generation (P) larvae number	10–15	15–20	15–20	10	10	20
Filial generation (F1 and F2) larvae number	40–60	40–60	40–60	25	25	20
Feeding frequency	Each 5 days	Each 3 days	Each 7 days	Each 10 days	Each 10 days	Each 10 days
Feeding method/ volume or weight	Solution (5 g/L)/5 mL	Solution (5 g/L)/2 Ml	Powder/2.5 mg	Powder/2.5 mg	Powder/2.5 mg	Powder/2.5 mg
Solution volume	500 mL	300 Ml	300 mL	340 mL	340 mL	340 mL for 500-militer plastic pots/500 mL for 1-L glass beaker
Replacement of test solution	No	Yes	Yes	Yes	Yes	Yes
Sediment	50 g	50 g	50 g	60 g	60 g	60 g
Replicates	3	3	3	3	4	4
Larvae sampling	P generation: 10 in all replicates F1 and F2 generation: 20–30 in all replicates	P generation: 10 in all replicates F1 and F2 generation: 20–30 in all replicates	P generation: 10 in all replicates F1 and F2 generation: 20–30 in all replicates	P generation: 10 in all replicates F1 and F2 generation: 20–30 in all replicates	10–13 larvae from the 4th replicate	20 larvae from the 4th replicate

Beakers with different material were evaluated considering the material available in the laboratory and distinct volume was dependent on: the solution volume to ensure a suitable depth of overlapping water and sediment and headspace to guarantee the swarming and mating development. The pots were firstly locked with tulle netting, but as the evaporation became an intense process, a plastic lid was used to prevent both evaporation and the scape of the midges. On the same line of reasoning, the replacement of test solution was proceeded order to compensate the evaporation.

The frequency of feeding was reduced in order to provide subsistence of the larvae and to avoid the appearance of fungi. Moreover, the number of larvae added from the second generation was decreased (from 40 to 60 to 20) to preventing intraspecies competition (food and mobilization) and avoiding mortality.

## *Chironomus sancticaroli* generation test

The condition that prolonged the duration of the test and allowed the best sampling of the organism was chosen as the best condition to proceed the *C. sancticaroli* generation test and is described as Assay: 6 (Table 1).

The tests are conducted with 4 replicates, using 1 L glass beakers or non-toxic 500-militer plastic pots, 340 mL or 500 mL of test solution (depending on the vessel chosen), 60 g of formulated sediment and feed with 2.5 mg of macerated fish food (Tetramin®). The pots have to be closed to prevent evaporation. The temperature varying between 23 and 27 °C and photoperiod of 12 h light / 12 h dark [3,9].

**Fig. 1:**
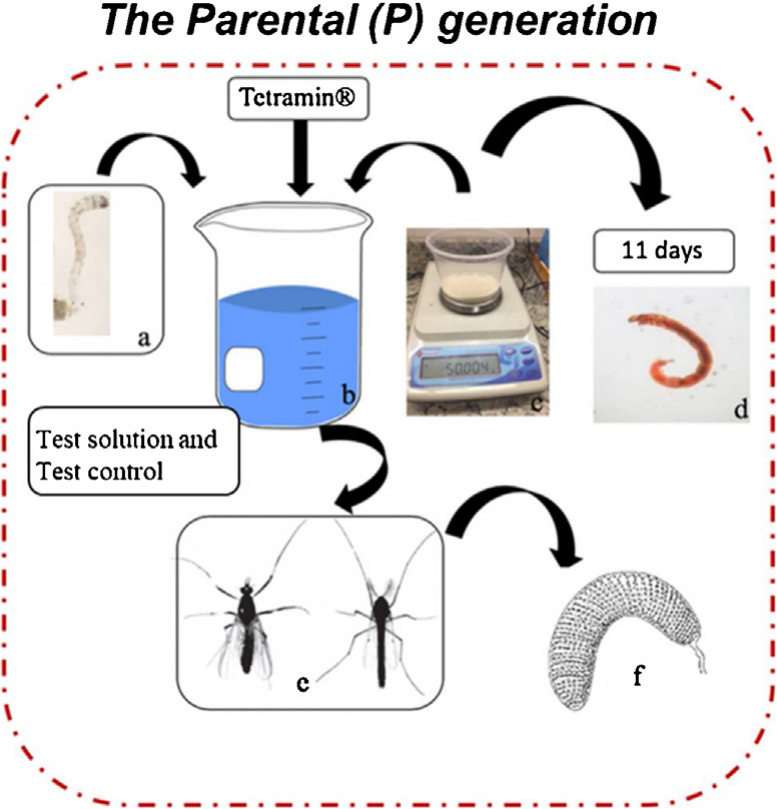
*C. sancticaroli* generation test procedure with P generation: a) I instar larvae of *C. sancticaroli*, b) glass vessel with 340 mL of test solution or deionized water, c) 60 g formulated sediment, d) IV instar larvae, e) adults emerged (female or male) and f) egg.

1) The Parental (P) generation (Fig. 1):

**Procedure a, b and c:** To start the test, an egg rope is used and placed in a Petri dish under slow and constant aeration until the egg mass larvae are fully developed (about 48 h). After larvae hatch the egg rope, 20 organisms are selected for each replicate in a Petri dish using a stereomicroscope and a glass Pasteur (arranged in lab watch glass). As recommended by Organization for Economic Co-operation and Development (OECD) [10], the addition of one replicate (from 3 to 4) can avoid larvae stress and allows to sample 20 larvae each time, used to observe the larvae length and buccal deformities endpoints. These organisms are placed in 1 L glass beakers or 500-militer plastic pots containing formulated sediment, test solution, feed with constant aeration and closed with tulle netting for glass beakers or with plastic lid for plastic pots to prevent evaporation. During the first 10 days after the test is set up, is necessary to observe the level of the test solution and, when necessary, replace it. On the 10th day, food is added to all replicates.

**Procedure d:** At the 11th day all alive larvae on the forth replicate is sampled and fixed on isopropyl alcohol to observe it development (larval length).

**Procedure e:** It is important that after 10 days of test the presence of adults is observed daily to ensure that the midges will be sample and to make any relocation when male and female were in different replicate. From the first emergence until all larvae become midge, the number of male and female have to be count (males are easily identified by their plumose antennae and thin body posture).

Emerging adults should remain in vessels until oviposition. If necessary, the female or male midge can be relocated in other replicate at the same test solution to provide swarming, mating and oviposition. After confirming the oviposition, the females are captured with an aspirator and placed in Eppendorf containing isopropyl alcohol for analysis of potential fecundity.

**Procedure f:** After the oviposition, the egg rope is carefully collected.

**Fig. 2:**
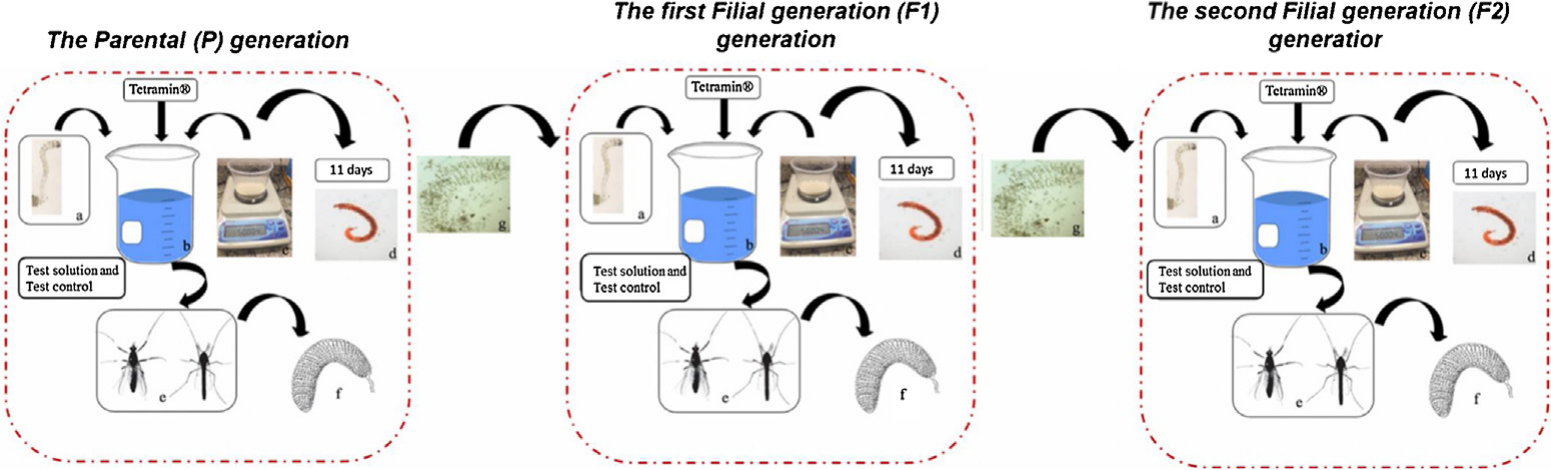
Sequential steps of the *C. sancticaroli* generation test: a) I instar larvae of *C. sancticaroli*, b) glass vessel with 340 mL of test solution or deionized water, c) 60 g formulated sediment, d) IV instar larvae, e) adults emerged (female or male), f) egg and, g) larvae hatching.

1) The filial (F1 and F2) generation (Fig. 2):

**Procedure a, b, c, d, e and f:** The procedures from a to f should be followed as described for P generation.

**Procedure g:** After the oviposition from the previous generation, the egg rope of each replicate should be placed in Petri dishes containing culture water and gentle aeration for 48 h until the larvae of the I instar are visible and start swimming. After this period, 4 replicates were assembled by adding 20 larvae in each, using stereomicroscope and glass Pasteur, with the same condition of the P generation (test solution), starting the next generation test (F1 or F2).

As a result of this study we could formulate a new methodology for *C. sancticaroli* generation test. It is an advanced because there is a necessity for a new ecotoxicological analysis that permits to conclude the lethal and sublethal effects of a substance and to verify environmental quality of water and sediment through generations. An important difference between our methodology and OECD n.233 [10] is that the hole life cycle of the organism occurs at the same vessel. So, it is possible have the control of number of larvae and adult’s emergence. This change minimizes disturbing and damaging of midges and, also allows the targeting of midges among the replicates, facilitating the swarming, mating and oviposition.

Possible endpoints to be analyzed:

This procedure allows the sequencing of the analysis of possible genetic mutations in organisms, such as alterations in larval development (absence or excess of teeth), alterations in adult wing morphometry, decrease in female wing length (fecundity) and on the body of the larvae (development). Following this conception of test, it is possible to analyze the following endpoints:

• Mortality: the number of organisms is counted at the number of the test, and the mortality ratio is calculated for each replicate;
• Larvae length: the larvae sampled are fix at isopropyl alcohol and placed on a sheet of glass above a graph paper. The length of the larvae is estimated in Image J software;
• Buccal deformities: the cephalic capsule is placed on a sheet of glass and analyzed with 10x magnification under an optical microscope. The presence of gap, addition or absence of teeth are considered buccal deformity;
• Adult emergence ratio: the number of adult midges was taken daily, and the ratio of male/female is calculated;
• Potential fecundity: the fecundity is estimated by the wing length following the methodology described by Trivinho-Strixino [6] (1980).

## Financial support

This research was supported by CAPES (Coordination for higher Education Staff Development) and CNPq (National Council of Technological and Scientific Development).

## Additional information

For the monitoring of the test it is suggested the daily annotation of the parameters: date; number of emerged females; number of emerged males; number of spawning; any change between vessel (if any adult needs to be changed in order to allow copulation, it should be noted as relocation (initial vessel - final vessel) (Table 2). Also, any observations on dead pupae, larvae, appearance of fungus and any other difference should be noted.

**Table 2:** Suggested table to be used during the *C. sancticaroli* generation test.

Test solution/Sediment sample			Generation	Start date	Final date
Date	Male	Female	Egg rope	Relocation	Observation
–	–	–	–	E.g. From replicate “x” to replicate “z”	E.g. 1 male was sampled; 1 female was escaped; …

## References

[1] Tassou K.T., Shulz R. (2009). Effects of the insect growth regulator pyriproxyfen in a two-generation test with Chironomus riparius. Ecotoxicol. Environ. Saf..

[2] Taylor E.J., Blockwell S.J., Maund S.J., Pascoe D. (1993). Effects of lindane on the life-cycle of a freshwater macroinvertebrate Chironomus riparius Meigen (Insecta Diptera). Environ. Contam. Toxicol..

[3] Fonseca A.L. (1997). Avaliação da qualidade da água na Bacia do Rio Piracicaba/SP através de testes de toxicidade com invertebrados.

[4] Fonseca A.L., Rocha O. (2004). Laboratory cultures of the native species *Chironomus xanthus* Rempel, 1939 (Diptera-Chironomidae). Acta Limnol. Bras..

[5] Corbi J.J., Trivinho-Strixino S., dos Santos A. (2008). Environmental evaluation of metals in sediments and dragonflies due to sugar cane cultivation in neotropical streams. Water Air Soil Pollut..

[6] Strixino G., Trivinho-Strixino S. (1985). A temperatura e o desenvolvimento larval de *Chironomus sancticaroli* (Diptera: Chironomidae). Rev. Bras. Zool..

[7] Novelli A., Vieira B.H., Cordeiro D., Cappelini L.T.D., Vieira E.M., Espíndola E.L.G. (2012). Lethal effects of abamectin on the aquatic organisms Daphnia similis, Chironomus xanthus and Danio rerio. Chemosphere.

[8] Viveiros W. (2012). *Chironomus sancticaroli* – do cultivo em laboratório ao ensaio ecotoxicológico com amostras ambientais de sedimento.

[9] Dornfeld C.B., Espíndola E.L.G., Fracácio R., Rodrigues B.K., Novelli A. (2006). Comparação de Bioensaios Laboratoriais e “in situ” Utilizando Chironomus xanthus na Avaliação da Toxicidade de Sedimentos do Rio Monjolinho (São Carlos, SP). J. Braz. Soc. Ecotoxicol..

[10] (2010). Test No. 233: Sediment-Water Chironomid Life-Cycle Toxicity Test Using Spiked Water or Spiked Sediment.

